# Lead Modulates *trans-* and *cis-*Expression Quantitative Trait Loci (eQTLs) in *Drosophila melanogaster* Heads

**DOI:** 10.3389/fgene.2018.00395

**Published:** 2018-09-20

**Authors:** Wen Qu, Katherine Gurdziel, Roger Pique-Regi, Douglas M. Ruden

**Affiliations:** ^1^Department of Pharmacology, Wayne State University, Detroit, MI, United States; ^2^Department of Obstetrics and Gynecology, Wayne State University, Detroit, MI, United States; ^3^Center for Molecular Medicine and Genetics, Wayne State University, Detroit, MI, United States; ^4^Institute of Environmental Health Sciences, Wayne State University, Detroit, MI, United States

**Keywords:** developmental lead exposure, *Drosophila melanogaster*, expression quantitative trait loci (eQTL), *Drosophila* synthetic population resource (DSPR), RNA-seq, toxicogenomics

## Abstract

Lead exposure has long been one of the most important topics in global public health because it is a potent developmental neurotoxin. Here, an eQTL analysis, which is the genome-wide association analysis of genetic variants with gene expression, was performed. In this analysis, the male heads of 79 *Drosophila melanogaster* inbred lines from *Drosophila* Synthetic Population Resource (DSPR) were treated with or without developmental exposure, from hatching to adults, to 250 μM lead acetate [Pb(C_2_H_3_O_2_)_2_]. The goal was to identify genomic intervals that influence the gene-expression response to lead. After detecting 1798 *cis-*eQTLs and performing an initial *trans*-eQTL analysis, we focused our analysis on lead-sensitive “*trans*-eQTL hotspots,” defined as genomic regions that are associated with a cluster of genes in a lead-dependent manner. We noticed that the genes associated with one of the 14 detected *trans*-eQTL hotspots, Chr 2L: 6,250,000 could be roughly divided into two groups based on their differential expression profile patterns and different categories of function. This *trans*-eQTL hotspot validates one identified in a previous study using different recombinant inbred lines. The expression of all the associated genes in the *trans*-eQTL hotspot was visualized with hierarchical clustering analysis. Besides the overall expression profile patterns, the heatmap displayed the segregation of differential parental genetic contributions. This suggested that *trans*-regulatory regions with different genetic contributions from the parental lines have significantly different expression changes after lead exposure. We believe this study confirms our earlier study, and provides important insights to unravel the genetic variation in lead susceptibility in *Drosophila* model.

## Introduction

### Expression QTLs (eQTLs)

One of the biggest challenges in biology is to understand how genetic variation alters gene expression, which is also known as genetical genomics (Mackay et al., 2009; Massouras et al., 2012; Lagarrigue et al., 2013). Genetics of gene expression has been studied in various species, such as maize (Schadt et al., 2003), yeast (Brem et al., 2002, 2005; Yvert et al., 2003; Bing and Hoeschele, 2005), roundworms (Francesconi and Lehner, 2014), flies (Gupta et al., 2007; Massouras et al., 2012; Zhou et al., 2016, 2017), mice (Schadt et al., 2003; Huang et al., 2009), and humans (Schadt et al., 2003; Mangravite et al., 2013; Zhang et al., 2014). Expression Quantitative Trait Loci (eQTL) analyses, which search for genomic loci that are responsible for the differential gene expression levels, has shed light on the genetic structure of transcriptional regulation. The first achievement in this field was seen in the budding yeast, where differential gene expression was shown to be segregated by parental genotypes (Brem et al., 2002).

Significant eQTLs are often categorized into two sub-groups: *cis-*eQTLs and *trans-*eQTLs. By their classical definitions, *cis-*eQTLs refer to genetic variants that affect a locus expression only on the same haplotype, while *trans-*eQTLs affect both haplotypes (Hirsch et al., 2003; Joo et al., 2014). Therefore, *cis-*eQTLs tend to be “local”—near the locus of the gene encoding the regulated transcript, while *trans-*eQTLs tend to be “distant”—away from the locus of the regulator (Hirsch et al., 2003; Joo et al., 2014). During the past several years, multiple *cis-*eQTLs were detected in human lymphoblastoid cell lines (Mackay et al., 2009; Pickrell et al., 2010; Mangravite et al., 2013). Several disease-specific *cis-*eQTLs were also detected, one of which proved the correlation between a statin-related eQTL for the gene *GATM* (glycine amidinotransferase), that encodes the rate-limiting enzyme in creatine synthesis, and statin-induced myopathy (Mangravite et al., 2013).

In contrast to the frequent identification of *cis-*eQTLs, fewer *trans-*eQTLs were identified, let alone disease-specific *trans*-eQTLs. A *trans*-eQTL hotspot is defined as one single location that is associated with the regulation of multiple genes, regardless of their transcript locations (Mangravite et al., 2013). *Trans-*eQTLs are more difficult to detect than *cis-*eQTLs since *trans* effects are often weaker than *cis* effects (Pierce et al., 2014). *Trans-*eQTL hotspots are emphasized in this paper because they are understudied in the field of toxicogenomics and because they are potentially toxin-induced master regulatory nodes of many downstream genes and pathways.

The existence of *trans-*eQTL hotspots was previously confirmed in budding yeast in 2003, where the gene *AMN1* (Antagonist of Mitotic Exit Network 1) was shown to *trans-*regulate a cluster of 12 downstream genes, irrespective of their transcript distances, and located throughout the yeast genome. *Trans-*eQTL hotspots are usually described as being eQTL in *trans-*regulatory factors, such as transcription factors or signaling proteins, but these types of eQTLs have been hard to identify outside of yeast, and require further study.

### Why lead?

Lead exposure has long been one of the most important topics in global public health. The major lead sources up until the 1970s, when they were restricted in the United States, were lead-containing paint and leaded gasoline. The phase-out of these two sources of lead in the US has resulted in dramatic reductions in mean blood lead levels (BLL); however, lead exposure from environmental contamination remains a major world public health issue (Dietrich et al., 2001; Maglott et al., 2005). It was reported by the World Health Organization (WHO) that lead exposure is predicted to account for 143,000 deaths per year throughout the world and it is considered as one of the highest burdens in developing countries (WHOteam, 2015).

The long-term effects of developmental exposure to lead on humans, especially on children, include damage to the nervous system, heart, bones, intestines, kidney, and reproductive system (Jedrychowski et al., 2011). In early 2012, the Centers for Disease Control (CDC) lowered the reference blood lead level for children and pregnant women from 10 to 5 μg/dl (Bellinger, 2013). Both the WHO and CDC have emphasized that no known level of lead is considered as “safe,” suggesting the irreversible danger of lead exposure (Bellinger, 2013; WHOteam, 2015). On a biological cellular level, the direct effects of lead toxicity include mitochondria damage, oxidative stress, disruption of calcium homeostasis, alteration of neurotransmitter release, altered function of neurotransmitter and receptors, and apoptosis.

Lead's ability to mimic as calcium makes it able to cross the blood brain barrier (BBB) (Bradbury and Deane, 1992). The effects of lead on neurotransmission include damage of the synapse, alteration of neurotransmitter receptors and apoptosis or necrosis in dopamine systems (Jabłonska et al., 1994). The molecular targets and genetic mechanisms of lead remain unclear, though N-methyl-D-aspartic acid receptors (NMDAR) have been believed to contribute to lead neurotoxicity at the synapse level (Baranowska-Bosiacka et al., 2012). NMDARs play a key role in synapse function and in the process of learning and memory. NMDARs are excessively stimulated by lead at toxic levels and this leads to excess calcium flow thorough the NMDARs, which could damage or kill the affected neurons (Marchetti and Gavazzo, 2005; Baranowska-Bosiacka et al., 2012).

To better understand how lead plays a role as a neurotoxin, and to identify lead-responsive genes that might be involved in lead neurotoxicity, we utilized the *Drosophila melanogaster* model to study the genetic effects of lead exposure during development. Our lab has already shown that *Drosophila* fed with 250 μM lead acetate in standard fly food, which results in lead levels of 50–100 μg/dl in tissue (Peterson et al., 2017), results in gene expression (Ruden et al., 2009), synaptic (He et al., 2009), and behavioral (Gupta et al., 2007) changes. We have previously found that lower lead levels in the food, 50 μM lead acetate, altered the uniformity of the synaptic match between the size of the motor neuron terminal and muscle fibers at larval neuromuscular junctions (Morley et al., 2003), and resulted in behavioral changes including courtship (Hirsch et al., 1995) and locomotor activity (Hirsch et al., 2003).

In a recent study on Detroit children, our laboratory has also shown that lead exposure could have multigenerational epigenetic effects (Sen et al., 2015). However, identifying the genetic mechanisms of lead induced neurotoxicity requires more detailed studies of gene regulatory networks. Our lab has previously used gene expression microarrays and eQTL analyses by comparing lead-treated whole *Drosophila* males to control ones and we have identified 12 genomic regions (5 in the control males and 7 in the lead-treated males) at 11 different loci (one was identified in both control and lead-treated males) that contain potential lead-responsive master regulatory genes (Ruden et al., 2009). While it was an intriguing result, this analysis only utilized 92 genotype markers, which was state-of-the-art at the time. In addition, each of the 12 genomic regions we identified as potential-trans-eQTL loci could only be restricted to a region of 5 centi-Morgans (cM), which hindered the ability to fine map the targeted genomic location and verify potential master regulatory genes.

To further validate the existence of *trans-*eQTL hotspots, our lab used another set of the *Drosophila* recombinant inbred lines (RILs), the *Drosophila* Synthetic Population Resource (DSPR), to conduct additional RNA-seq expression analyses on *Drosophila* heads instead of whole bodies. In this study, we used RNA-seq and focused on genomic information on 11,768 genomic markers (King et al., 2012b). Each sample from the DSPR was a mosaic of eight parental strains, which were from different geographic locations and should include a large swath of genetic variance. By using this information, we could restrict the regulatory genomic regions within 10 kb. In this paper, we present the results of these findings and provide further validation of the existence of lead-responsive *trans-*eQTL hotspots.

## Materials and methods

### Genotype data

The eight founder strains of *Drosophila* Synthetic Population Resource (DSPR) and their recombinant inbred lines (RILs) were kindly provided by Dr. Stuart Macdonald from the University of Kansas and Dr. Anthony Long from the University of California, Irvine. The RILs were started with eight founder strains, A1–A8 that were of diverse geographic origins and may include a great deal of the genetic variation in the *Drosophila* species (King et al., 2012b). Strains were first intercrossed, A1 was crossed with A2, A2 was crossed with A3, and this went on until A7 was crossed to A8 (King et al., 2012b). Next, 10 F1 flies per genotype per sex were mixed altogether and continued to produce offspring (King et al., 2012b). Until the 50th generation of crossing, offspring were separated and another ~25 generations of sibling inbreeding made the finished DSPR “A2 subpopulation,” consisting of ~800 RILs that each contain only ~1% of the heterozygous founder genotype (King et al., 2012b).

The DSPR constructed 96-plexed restriction-site associated DNA (RAD) libraries, which further resulted in the revelation of 10,275 SNPs (King et al., 2012b). They used the hidden Markov model (HMM) to convert the SNP data to estimate the probability of the underlying founder genotype for the *Drosophila* genome (genotyping error rate: 0.5%) (King et al., 2012b). Since all RIL samples are mostly homozygous and they have in total eight parents (marked as A1–A8), there are at most eight possible genomic origins for any genomic position. The *Drosophila* genome (only chromosome X, 2, and 3; chromosome 4 was excluded due to lack of genomic information from the DSPR group) was divided into 11,769 10 kb genomic segments, resulting in 11,768 markers at the junction point. The genotype dataset at the DSPR website shows the founder name of each of the 11,768 markers for all the samples (http://wfitch.bio.uci.edu/~dspr/).

### Sample preparation

All the fly stocks were reared at 25°C in 35 ml vials containing 10 ml of standard *Drosophila* medium. To mimic lead poisoning, the medium was mixed to a final concentration of 250 μM PbAc [Pb(II)(C_2_H_3_O_2_)_2_] for lead-containing medium or 250 μM NaAc [Na(C_2_H_3_O_2_)] for control medium. This makes the *Drosophila* brain contain 50–100 μg/dl lead content (Peterson et al., 2017). Our lab has long been using NaAc as the control for PbAc for nearly a decade, and was recommended by leaders in the lead toxicology field (Ruden et al., 2009). Also, 250 μM PbAc was considered as a mild dosage for *Drosophila melanogaster* and there was no immediate lethality upon lead poisoning in this study. Previous papers also mentioned that the survival rate to adulthood was not affected by the 250 μM lead exposure (Cohn et al., 1992). Recently, the Mackay and Anholt laboratories found that developmental time and viability are not generally affected in most Drosophila strains until PbAc concentrations are at 500 μM or higher (Zhou et al., 2016).

In our experiments, 79 randomly selected DSPR samples were fed, from egg to adult, either control food or lead-containing food until the heads were harvested when the adults were 3–7 days old. Fifty heads from each strain were manually collected by tweezers and immersed in RNAlater® solution to stabilize and protect the cellular RNA. The heads were collected in the morning around 10 a.m. We did not have any technical head or biological replicates, since we wanted the maximum inclusion of the RILs. Fifty heads of adult male flies (5–10 days old) in each of the 79 strains were collected and TruSeq™ Cluster RNA sample prep kits from Illumina were used to prepare the samples. One micrograms of RNA was used after RNA isolation. The High Sensitivity D1K ScreenTape™ on the Agilent TapeStation™ instrument and quantitative PCR on the QuantStudio™ 12K Flex were used to make sure the quality of the library was adequate. RNA expression analyses were performed with 50-cycle paired-end RNA-seq on the HiSeq2000™ instrument from Illumina. General read quality was verified using FastQC™ (Hirsch et al., 1995). The average coverage is 23 million read pairs, and the RNA-seq data are available on the NCBI GEO accession: GSE83141.

### Expression profiling

Tophat2™ (V2.0.8) was used to map reads against the known *Drosophila melanogaster* (UCSC/dm^3^) transcriptome (Kim et al., 2013). The transcript assembly tool Cufflinks™ and differential expression tool Cuffdiff™ were utilized for gene discovery and comprehensive expression analysis of RNA-seq data (Trapnell et al., 2012). After the Cufflink™ pipeline, we assembled all the expression data and quantile normalized to the overall average empirical distribution across all samples first, then across all genes. When doing the differential expression analysis, we used the RIL information as a covariate (Y ~ treatment + RIL). Gene Ontology (http://geneontology.org/) (Kent et al., 2002; Young et al., 2010) was used for the GO enrichment analysis for the differentially expressed genes upon lead exposure and GP categories of “Molecular Function” and “Biological Process” were selected.

### Genome-wide eQTL mapping

A data analysis R package called DSPRqtlDataA (http://wfitch.bio.uci.edu/~dspr/index.html), provided by the DSPR group (King et al., 2012b), was used to extract the genotype dataset indicating the genomic origin at 10,768 loci for each sample. Like what the DSPR group did, we performed a multiple regression—regressing gene expression profiles on the eight additive genotype probabilities with zero covariate.

$$\begin{array}{l} {\text{H}}_{0}:\text{Y}=\mu +\varepsilon \\ \text{H}:\text{Y}=\mu +\sum G_{i}+\varepsilon \end{array}$$

Where μ is the grand mean, *G*_*i*_ is the ith parental genotype probability.

The LOD score, which is the logarithm of odds base 10, was used to quantify the likelihood of association between 10,768 genomic locations and 13,381 gene expression profiles among 79 paired samples (one control and one lead-treated). It compares the likelihood of obtaining the test data if the two loci are indeed associated to the likelihood of observing the same data purely by chance. Positive LOD score favors the presence of correlation.

$$\begin{array}{l} \text{LOD score}={\text{log}}_{10}\left({\text{Likelihood of H}}_{1}\right)-{\text{log}}_{10}\left({\text{Likelihood of H}}_{0}\right) \end{array}$$

After obtaining the LOD score between each genomic location and each gene expression level, we determined the significance threshold for each gene via 1,000 permutations on its expression levels.

For each of the genes, the expression levels for all samples were extracted, randomly shuffled, and a new LOD score was calculated for all loci based on the shuffled expression list. This process was repeated for 1,000 times and 1,000 LOD scores were produced for each gene. Based on the 1,000 permutation, an empirical null distribution could be generated and an eQTL *p*-value for the gene/locus association could be calculated accordingly. This permutation-based *p*-value is defined as the quantile of the observed LOD score on the empirical permutation based null distribution.

$$\begin{array}{l} \text{p}-\text{value for gene x=}\frac{\begin{array}{l} \text{numbers of permutations for whose LOD score}\\ \leq \text{ observed LOD score } \end{array}}{\text{total number of permutations }(\text{=1000})} \end{array}$$

After obtaining all the eQTL *p*-values, we defined significant eQTLs as *p*-value ≤ 0.05, defining *cis-*eQTL genes as the ones have significant associations with at least one genomic location within 1 cM geographic distance and *trans-*eQTL genes as those have significant associations with genomic locations outside of 1 cM. The “qvalue” function in R was used to transform *p*-value into FDR. *p*-value ≤ 0.05 is equivalent to FDR ≤ 11% for the *cis-*eQTLs. The *cis-*eQTLs are adjusted for multiple testing, however few *trans-*eQTLs would survive this adjustment.

As the King et al. pointed out, we might not have the power to detect individual *trans-*eQTLs (King et al., 2012a), that is why we proceeded to look for loci that may have *trans-*eQTL hotspots with an enrichment-like type of analysis for hotspots with an unusually high number of *trans-*eQTL (loci with excess of low *p-*values < 0.05 at distant regions). For the *trans-*eQTL hotspot we calculate the enrichment “hotspot *p*-value” that seeks to answer the question of whether the number of genes associated with a given locus is much higher than what would be expected by chance. To answer this question, we used a statistical measure: the number of genes associated with the locus (# eQTL *p*-value < 0.05, vertical band on **Figure 2**). To generate an empirical null distribution for this number, we permuted 1,000 times the eQTL results across the different loci (i.e., vertical bands). This empirical distribution is then used to calculate the permutation based “hotspot *p*-value.”

Given that multiple nearby genomic locations had similar numbers of associated genes and were all considered as significant, we wondered whether these hotspots were indeed genetically separable. To test this, we tested the associations of nearby *trans-*eQTL hotspot peaks by multiple regression and combined hotspots that have similar influence over the downstream genes. According to this threshold, we re-categorized the *trans-*eQTL hotspots (Table S1) and had 14 *trans-*eQTL hotspots as a result. The *trans-*eQTL hotspots were presented in the format of a region, which had a starting location, an ending location and a peak location, which has the most associated genes (details in Table S1).

After the discovery of these hotspots, surrogate variable analysis (SVA) was used to control for potential confounders when analyzing *trans-*eQTLs (Pickrell et al., 2010), and the following model was used to identify eQTLs with Gene-by-Environment interaction:

$$\begin{array}{l} \text{H}0:\text{Y}=\mu +\text{S}+\sum G_{i}+\text{E}+\varepsilon \\ \text{H}:\text{Y}=\mu +\text{S}+\sum G_{i}+\text{E}+\sum G_{i}*\text{E}+\varepsilon \end{array}$$

where E represents two conditions: control or lead-treated, and S represents the surrogate variables.

### Common motif search by Genomatix™

Promoter sequences of the 89 anticipated downstream genes at Chr 2L: 6,250,000 were obtained by using the software program Gene2Promoter (Genomatix™ software package used for retrieval and analysis of promoters) at default settings, 500 bp upstream of the first TSS and 100 bp downstream of last TSS. CoreSearch™ was then used by input of these sequences in FASTA format to screen for any unknown common motifs among the sequences (Wolfertstetter et al., 1996). It creates a novel position weight matrix from the input sequences. Tomtom™ was also performed to search for matches with the existing pool of motif databases (Gupta et al., 2007). Interactions browser was used in search for protein-protein interactions (http://flybase.org/cgi-bin/get_interactions.html) (Wolfertstetter et al., 1996).

## Results

### Differential expression caused by chronic lead poisoning

In order to further understand and validate the *trans-*eQTL hotspots detected in our 2009 microarray paper on the neurotoxicity of lead in *Drosophila* (Ruden et al., 2009), we collected RNA-seq data from the heads of 79 recombinant inbred lines (RILs) selected from the *Drosophila* Synthetic Population Resource (DSPR) (King et al., 2012b). The DSPR was composed of a panel of ~1,600 *Drosophila* lines (King et al., 2012b). The lines were initiated with eight parental strains A1–A8 that are from different geographic origins and should include a good mix of genetic variation in the *Drosophila* species which were intercrossed for 50 generations and then inbred for another 25 (King et al., 2012b). We randomly selected 79 lines from the synthetic population and offspring were fed, from egg to adult, either control food (containing 250 μM NaAc) or lead-treated food (containing 250 μM PbAc). Fifty heads of adult male flies (5–10 days old) in each strain were collected and RNA expression analyses were performed (see section Materials and Methods). Thus, we had 79 control and 79 lead-treated RNA-seq samples from heads from the same lines that we could analyze for differentially expressed genes.

Dramatic effects were seen on gene expression profiles after lead poisoning: 2,698 among the 13,381 expressed genes, including 68 exhibiting over 50% change in expression levels. [20%, false discovery rate (FDR) < 0.0001, 0.214 ± 0.223 mean absolute log_2_ change ± s.d.] (Figure S1, see Methods). Among the responders, 2,038 genes were upregulated after lead treatment, among which nervous system development and neurogenesis were the topmost enriched gene ontology (GO) categories (Figure S2). On the other hand, among the 660 genes downregulated upon lead exposure, developmental growth and synaptic target recognition were among the most enriched GO categories (Figure S2). These results were consistent with our expectation, since only *Drosophila* heads were collected on sample preparation and the neurotransmitters at the synaptic levels has long been considered as the main targets for lead neurotoxicity (Baranowska-Bosiacka et al., 2012). Genes that are metal responders, like *Metallothionein B, C, D*, and *E*, and neuro-related genes like *Nacalpha, dhd*, and *RpS5b* were among the strongest responders. *N-Methyl-D-Aspartate* (*NMDA1* in *Drosophila*) and its Receptors (*NMDAR1* and *NMDAR2*), previously identified as lead target at the synapse level (Marchetti and Gavazzo, 2005; Baranowska-Bosiacka et al., 2012), were also among the differentially expressed genes [*NMDA1*: log_2_FC = 7.809, FDR = 0.014; *NMDAR1*: log_2_FC = 1.004 (i.e., ~2-fold increase), FDR = 0.005; *NMDAR2*: log_2_FC = −1.150, FDR = 0.004].

### Identification of *cis-* and *trans-* eQTLs

After identifying genes that were affected by lead treatment, we worked on identifying expression quantitative trait loci (eQTLs)—the genomic region with genetic variants that affect gene expression levels. In most eQTL studies (Ruden et al., 2009; Mangravite et al., 2013), SNPs were used to represent the genotype. However, in our study, each sample was a mosaic of the eight parental lines (A1–A8) (details in Materials and Methods) and we used directly the information provided by the DSPR—the genetic contribution by the parental genotypes, which means the parental line a certain genomic region of the offspring was inherited from. With this type of genotype information, the eQTL was defined as a genomic location where gene expressions were associated with differential parental contribution.

The readily available DSPR R package was designed for single gene eQTL search (http://wfitch.bio.uci.edu/~dspr/Tools/Tutorial/index.html); therefore, we re-structured it to allow multiple gene eQTL searches (see section Materials and Methods). By using the newly modified R program, we computed the LOD score to quantify the likelihood of strong association between genomic locations and gene expressions. One thousand permutations were run to estimate the threshold of statistical significance (see section Materials and Methods).

**Figure 1:**
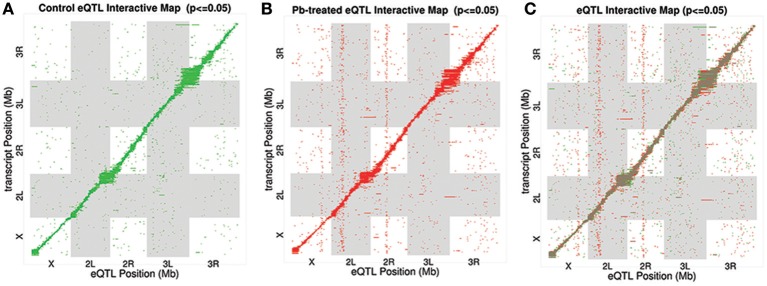
eQTL Map. All significant associations were shown in an eQTL map with eQTL locations (genomic loci) on x-axis and transcript locations (gene loci) on y-axis. **(A)** Associations for control samples only. Each of the green dots indicates a significant association between the corresponding eQTL location and the gene at the transcript location. **(B)** eQTL Interactive Map for lead-treated samples only. Each of the red dots indicates a significant signal. **(C)** eQTL Interactive Map combining both control and lead-treated samples. Shared significant signals were marked as brown, with lead-specific signals as red and control-specific ones as green.

After searching for all possible associations among 13,381 gene expression profiles against 11,768 genomic locations, we visualized the entire significant associations with an eQTL map (Figure 1A for control panel, Figure 1B for lead-treated panel and Figure 1C for the merged panel). Each of the colored dots represents one significant correlation between the genetic location displayed on the x-axis and the gene on the y-axis (significance at 0.05 for 1,000 permutation). There was a prominent diagonal band in both control and lead-treated map. It showed that transcript locations of this band of genes were equal to the eQTL location, thus the cluster of genes belong to *cis-*eQTLs. On the other hand, there were also some distinguished vertical bands, indicating any one of these genomic regions was associated with genes across the entire chromosome. These genomic loci with a high density of eQTLs are usually called *trans-*eQTL hotspots (Joo et al., 2014; King et al., 2014) or *trans-*eQTL bands (Rockman and Kruglyak, 2006). In total, we got six control and eight lead-treated *trans-*eQTL hotspots (see Method for more details, **Figure 3**, Table S1). Among them, ten were lead-sensitive hotspots (Table S1).

**Figure 2:**
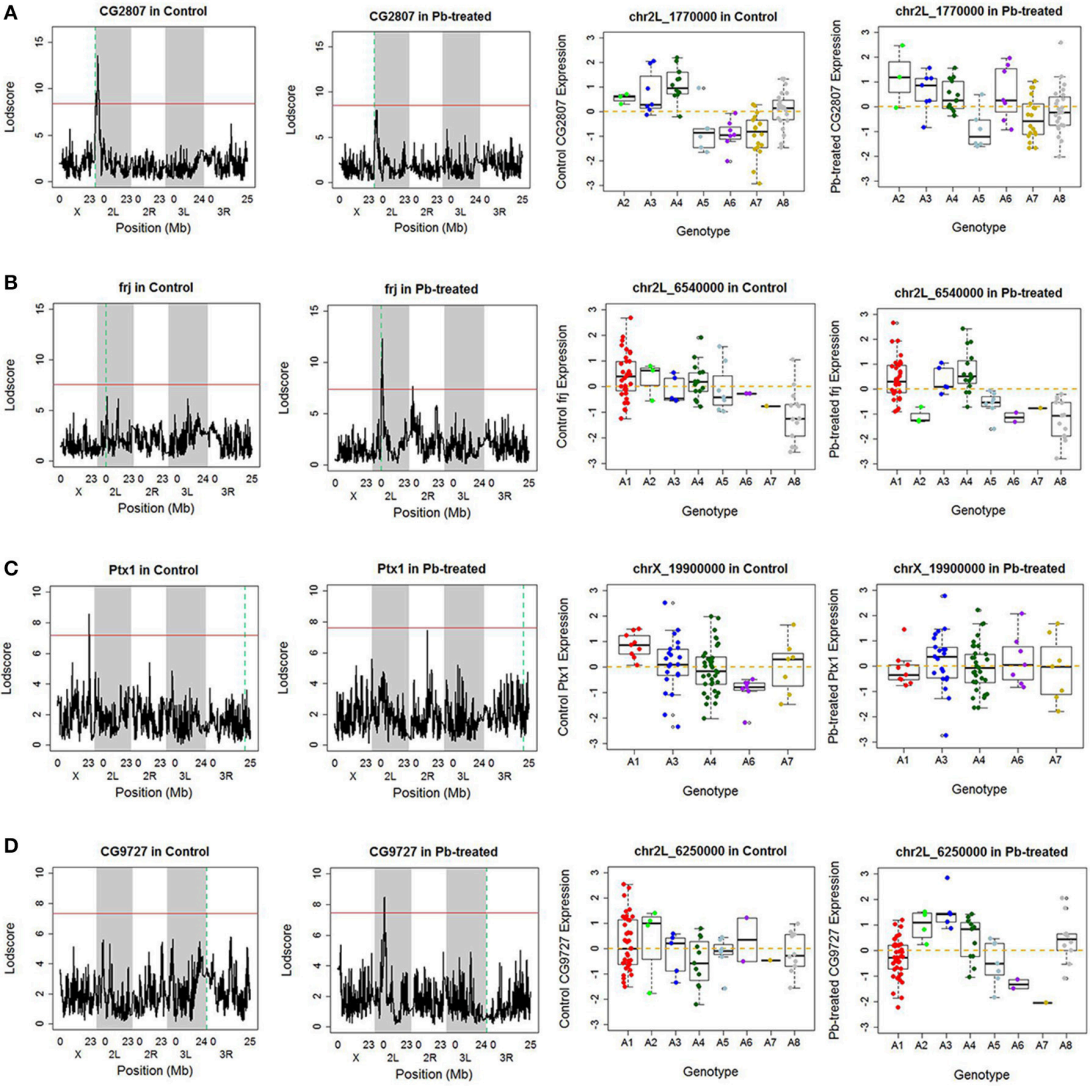
Examples of *cis-* and *trans-* eQTLs. **(A)** One example of control-specific *cis-*eQTL. In the left two panels, the x-axis represents the *Drosophila* genomic locations and y-axis represents the LOD score of the gene. The red horizontal line indicates the threshold for *p*-value to be 0.05 after 1,000 permutation test. The green dash vertical line indicates the location of the gene. If it overlaps with the peak, which suggests strong correlation between the gene and the corresponding location, it is referred as a *cis-*eQTL, meaning the regulator is near the downstream gene. Since this phenomenon only occurred in control data but not in lead-treated one, this genomic location Chr 2L: 1,770,000 is a control-specific *cis-*eQTL for gene CG2807. In the right two panels, association of the Chr 2L: 1,770,000 location, which has the highest LOD score in control samples, with quantile normalized CG2807 expression levels following control (not significant) and Lead-treated (*p*-value < 0.001). Samples originally from A2, A3, and A4 parental lines exhibited higher expression levels, while samples from A5, A6 and A7 parental lines showed lower expression levels in control. After lead was introduced, the phenomenon disappeared. **(B)** One example of Lead-specific *cis-*eQTL. **(C)** One example of control-specific *trans-*eQTL. **(D)** One example of Lead- specific *trans-*eQTL.

Along the diagonal, we detected 1798 *cis-*eQTLs (FDR ≤ 11%) (Figure S3A). Among the genes with *cis-*eQTLs, 997 genes were shared among control and lead-treated, along with 405 control-specific and 396 lead-specific (Figure S3A). One example of the control-specific *cis-*eQTL was shown in Figure 2A. In this example, left two panels showed all the LOD scores for the gene CG2807 at each of the 11,768 evenly divided genomic locations for both control and lead-treated status. Therefore, the high peak in the control panel indicated strong association with the corresponding genomic location on the x-axis but this signal disappeared after lead treatment (Figure 2A, second to the left panel). Also near the strongest peak, we found it does overlap with the gene location (green dashed line) and this indicated that the gene CG2807 is not only a control-specific eQTL but also a *cis-*eQTL.

To further explore the parental contribution of the genomic location at the highest peak in control, we sub-grouped the gene expression levels according to their parental genotypes at this peak location (Chr 2L: 1,770,000) and used a boxplot to show their expression pattern (Figure 2A, right two panels). From the figure, samples originally from A2, A3, and A4 have significantly higher expression levels than samples from A5, A6, and A7 in control, while this phenomenon was greatly reduced in lead-treated samples. This allelic heterogeneity was also widely seen in DSPR female head eQTL study (King et al., 2014). In addition to the control-specific *cis-*eQTLs, there is an example of lead-specific *cis-*eQTL in Figure 2B.

**Figure 3:**
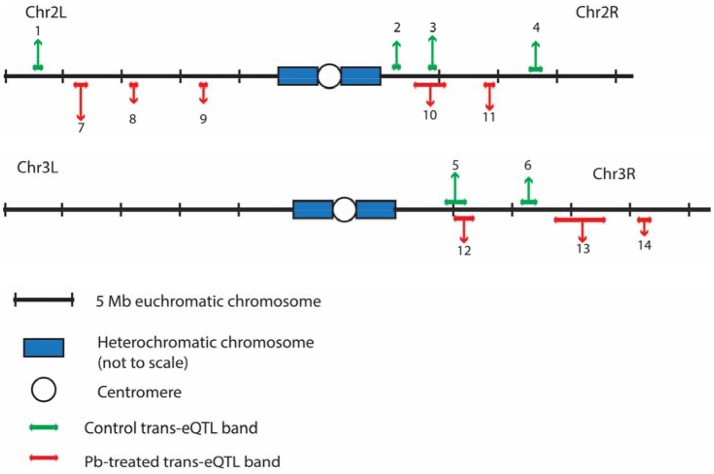
The distributions of *trans-*eQTL hotspots among the *Drosophila* genome. Six for control (green, above the genomic axis) and 8 for lead-treated (red, under the genomic axis) *trans-*eQTL hotspots were detected in total. Chr 4 and heterochromatic chromosomes were excluded due to lack of genomic information from the DSPR group. Chr X has no *trans-*eQTL hotspots. All the *trans-*eQTL hotspots were numbered and the length of each arrow roughly represents the number of the associated genes. Details about these hotspots were shown in Table S1.

Outside of the main diagonal, we will still have 4376 potential *trans-*eQTLs (Figure S3B) (1,000 permutation *p*-value < 0.05). Among the 4,376 genes with *trans-*eQTLs, 1,851 genes were shared among control and lead-treated, along with 1,058 control-specific and 1,467 lead-treated (Figure S3B, one examples of control-specific *trans-*eQTLs in Figure 2C and one for the lead-specific *trans-*eQTLs in Figure 2D). Few *trans-*eQTL associations would survive adjustment of multiple test correction. King et al. suggested that the power to map a 10% QTL with 100 DSPR lines is potentially about 15% for recombinant inbred lines (RILs) and less than 5% for pA-pB cross F_1_ hybrids (King et al., 2012a). Therefore, it is challenging to map *trans-*eQTLs, even if their effects are relatively large, with 79 RILs. To attempt to solve the problem, we used the recombinant inbred lines (RILs) rather than F_1_ hybrids, and used 1,000 permutations to obtain a well calibrated *p*-value. Rather than focusing on individual *trans-*eQTLs that would not survive multiple hypothesis correction, we focused on bands where *p*-values < 0.05 were enriched. Along the diagonal we can see the *cis-*eQTLs, but we also see vertical bands that may represent *trans-*eQTL hotspots. Here, we developed a secondary analysis that focuses on locus that may be a *trans-*eQTL for many genes and we found 14 *trans-*eQTL hotspots as a result (Figure 3, Table S1).

### Genetic dissection of the *trans*-eQTL hotspots

**Figure 4:**
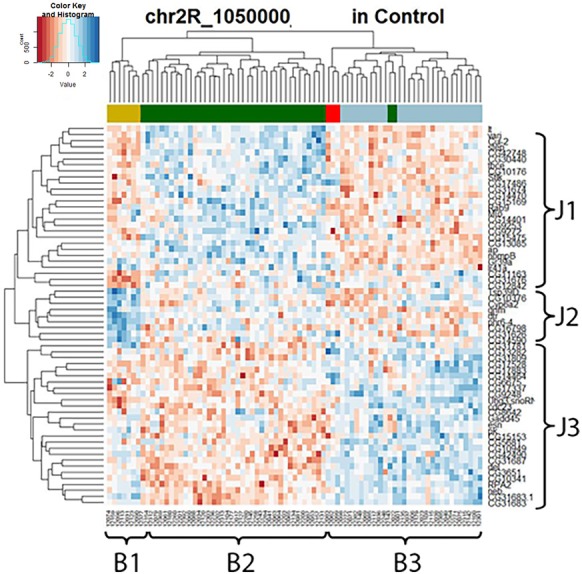
Stable *trans-*eQTL hotspot at Chr 3R: 5,580,000 (*p-*value < 0.05 at 1,000 permutation threshold). Hierarchical clustering analysis was done according to the expression profiles of the Chr 3R: 5,580,000 associated genes (*p*-value < 0.05). Plotted by using the lead-specific *trans-*eQTL, the bottom list indicates all the sample names and the right list indicates all the associated genes. The color-coded bar above the heatmap and below the dendrogram indicates the original parent of each sample listed at the bottom for this specific location. Color legend in the color-coded bar: red: A1, green: A2, blue: A3, dark green: A4, light blue: A5, purple: A6, gold: A7, darkgray: A8.

To further explore the mechanism of the *trans-*eQTL hotspots, we first looked at the stable *trans-*eQTL hotspots, meaning they were present in both control and lead-treated (one example in Figure 4). A hierarchical clustering heatmap (Eisen et al., 1998) was used to display the expression patterns of all the associated genes (Figure 4). This type of clustering analysis uses statistical algorithms to re-order genes according to the similarities of their expression patterns. In Figure 4, all associated genes with the locus of Chr 3R: 5,580,000 were arranged into three groups (J1, J2, and J3) for genes (right list) and another three groups (B1, B2, and B3) for samples (bottom list). Interestingly, the segregation of samples according to the expression pattern overlaps with the genetic contribution of the parental genotypes (the color-coded bar above the heatmap): samples originally from A3 (blue) showed lower J1+ J2 expression pattern and higher J3 expression pattern, while samples from A4 (dark green) had the opposite pattern.

Not only did we find the correlation between expression traits and parental contribution at the stable *trans-*eQTL hotspots, but also in lead-responsive ones. Here, we used the one that located at Chr 2L: 6,250,000 and contained the most associated genes as our example for lead-sensitive *trans-*eQTL hotspots in the following study. We wanted to focus this paper on the *trans-*eQTL at Chr 2L: 6,250,000 because the other *trans-*eQTLs are either less robust or located near centromeric, which are non-recombinogenic regions of the genome.

**Figure 5:**
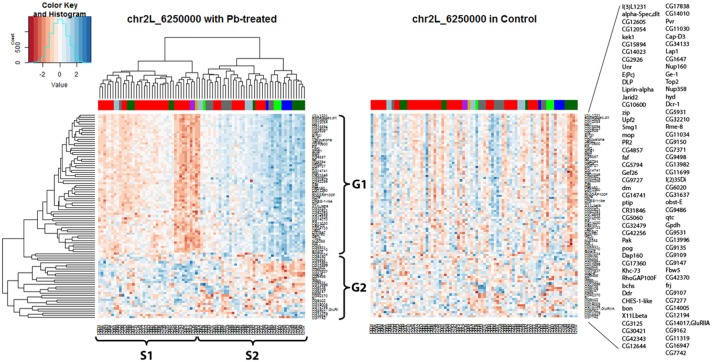
*Trans-*eQTL hotspot at Chr 2L: 6,250,000 (*p*-value < 0.05 at 1,000 permutation threshold). Hierarchical clustering analysis was done according to the expression profiles of the Chr 2L: 6,250,000 associated genes (*p*-value < 0.05). On the left heatmap plotted by using the lead-specific *trans-*eQTL, the bottom list indicates all the sample names and the right list indicates all the associated genes. The right heatmap was created by keeping the order of the sample names and associated gene names in the lead-treated plot on the right but replacing with control expression data. The expression patterns formed in lead-treated data disappeared in control data, suggesting this *trans-*eQTL hotspot could only be observed in expression levels after lead exposure. Color-coded bar above the heatmap and below the dendrogram indicates the original parent of each sample listed at the bottom for this specific location. Color legend for the color-coded bar: red: A1, green: A2, blue: A3, dark green: A4, light blue: A5, purple: A6, gold: A7, darkgray: A8.

Hierarchical clustering analysis was used again to present expression data graphically (Figure 5) and it showed that all the hotspot-associated genes were divided into two groups (G1, G2) and all the samples were divided into two groups (S1, S2) according to the nearby dendrograms. It appeared that genes from G1 exhibited lower expression levels in sample group S1 but higher in S2, while genes from G2 presented the opposite phenomenon. With the help of the color-coded bar on top of the heatmap, a segregation was shown among some of the samples based on their original parents: the expression pattern of samples from A2 (green) and A3 (blue) was in contrast with that of samples from A6 (purple) and A7 (gold). However, not all parents show unique influences on downstream genes, such as A1 (red) and A4 (dark green). This suggested that different strains of *Drosophila* species might respond differently to lead exposure and this was reflected by regulation of one key eQTL locus and its downstream gene expression levels. Compared with the lead-specific *trans-*eQTL hotspot containing 89 associated genes, only 28 associated genes were observed with the same genomic locus by using control expression data. If we replaced the heatmap (Figure 5 left panel) with control data but keeping the order of gene list and sample list same as the lead data, we noticed an entire disruption of the expression pattern present after lead exposure (Figure 5 right panel). This confirmed this hotspot at Chr 2L: 6,250,000 was only present after lead exposure.

In order to take a deeper look at the genes associated with the Chr 2L: 6,250,000 genomic location upon lead exposure, we searched for their GO enrichment categories (Attrill et al., 2015). Genes could be categorized into five groups: neuro-related, metal-related, response to stimuli and immune system, other metabolic processes, and unknown function (Table S2A). We noticed that genes in G1 were mainly related to neuronal function (18 out of 61, 30%), while genes in G2 were mostly metabolic processes (18 out of 28, 64%) (Details in Table S2A). We also recognized that genes in G1 (52 out of 61, 85%) were lead-specific eQTLs at Chr 2L: 6,250,000 (Table S2B, examples in Figure S2D, Figures S4A,B), while genes in G2 (22 out of 28, 78%) were more likely in closer proximity of the eQTL locus and were stable eQTLs (Table S2B, examples in Figures S4C,D). Among the rest of the signals, a few were Pb-specific eQTLs (Table S2A, one example from G1 in Figure S4E and one example from G2 in Figure 2B).

It has long been proposed that a transcription factor is a natural candidate for being the regulator of the *trans-*eQTL hotspots (Yvert et al., 2003). It has been hypothesized that the eQTL location may have influence over the affinity of a certain linked transcription factor and the transcription factor has multiple associations with downstream genes. This hypothesis serves as a perfect candidate explanation for *trans-*eQTL hotspots. However, it has been controversial ever since and not many studies have discussed about it. Yvert et al. (2003) mentioned that few *trans* variations have strong correlations with known or predicted transcription factors in their yeast research. In our case, we searched for common nucleotide motifs of the downstream genes at the *trans-*eQTL hotspot. Promoter sequences of all the 89 downstream genes at Chr 2L: 6,250,000 were extracted by using Gene2Promoter function (a tool to retrieve promoter sequences from the genome, see Methods) from the Genomatix™ Computer software suite (GmbH, 1998) and AAAAAYA (Y: C or T) was the most common motif generated after searching among the retrieved promoter sequences by using another Genomatix™ function CoreSearch™ (Figure S5). We also used Tomtom™ software for quantifying similarity between query motif and motifs from the existing databases to see whether this identified motif would match with any of the previously discovered ones (Gupta et al., 2007). It turned out that *hunchback* (*hb*) has shared motif with the AAAAAYA (*p*-value = 8.11e-04, Figure S5D). The *hb* gene, which encodes a Zn-finger transcription factor in the gap-gene class, locates at Chr 3R—one of its developmental functions involves neuroblast fate determination (Isshiki et al., 2001; Tran et al., 2010; Attrill et al., 2015). *hb*, as a transcription factor, has been shown to be necessary for regulation of the first-born glial cell fates, leading a sequence of transcription factors at the cell fate specification stage (Isshiki et al., 2001). Interestingly, the *hb* locus was not detected to be an eQTL by itself (Figures S5E,F). There were also no known protein-protein interactions between *hb* protein and any of the proteins encoded by the associated genes at the *trans-*eQTL hotspot.

Our next consideration was to verify the existence of the *trans-*eQTL hotspot at Chr 2L: 6,250,000. For eQTL analysis, one of the major concerns is expression heterogeneity (EH) (Pickrell et al., 2010; Joo et al., 2014). We used Surrogate Variable Analysis (SVA) to test whether the *trans-*eQTL hotspot could still be considered as significant after controlling for EH (see Methods) (Pickrell et al., 2010). The *trans-*eQTL hotspot at Chr 2L: 6,250,000 locus was still one of the strong peaks after SVA processing (Figure S6). This indicated that the lead-sensitive *trans-*eQTL hotspot could be considered as a true positive result.

In addition to the SVA processing, the best way to validate a *trans-*eQTL hotspot is by using another set of lead-treated expression data and see if similar expression patterns exist in the independent dataset. Our lab does have another set of lead-treated *Drosophila* gene expression data in the form of gene-expression microarray data that we published from whole-male lead-exposed *Drosophila melanogaster* that we published in 2009 (Ruden et al., 2009). In contrast to the eight-way RNA-seq data we currently have, the microarray dataset was a two-way eQTL analysis, meaning the samples were originally from two parents (comparison of the two experimental designs is in Table S3).

**Figure 6:**
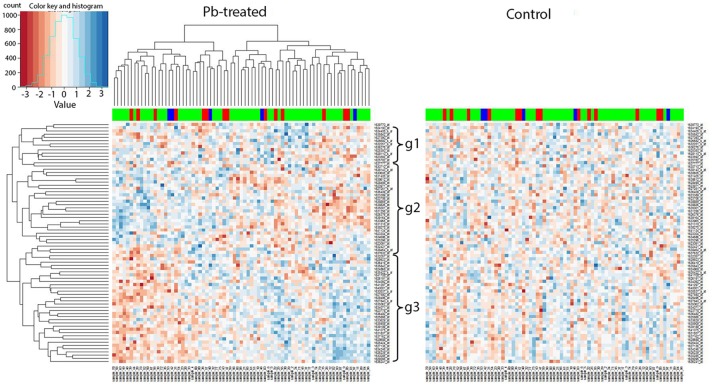
*Trans-*eQTL hotspot at 27E (nearest to Chr 2L: 6,250,000). Expression levels of candidate downstream genes detected with RNA-seq were extracted from the microarray data and hierarchical clustering analysis was done afterwards. In the left panel (lead-treated), genes could be roughly divided into three groups g1, g2, and g3. Most genes from g1 and g3 belong to G1 from the RNA-seq data, while most genes from g2 were the same as G2. The right heatmap was created by keeping the order of the sample names and associated gene names in the lead-treated plot on the right but replacing with the control expression data. The expression patterns formed in lead-treated data disappeared in control data, suggesting the genes forming expression patterns could only be observed in expression levels after lead exposure. The color-coded bar above the heatmap and below the dendrogram indicates the original parent of each sample listed at the bottom at this specific location. Color legend in the color-coded bar: red: Oregon R (ORE), green: Russian 2B (2B), blue: heterozygous. No segregation based on the parental origin was seen, suggesting these two parental lines do not differ in expression levels and this also explains why this has not been detected as a *trans-*eQTL hotspot in the microarray data.

We applied our current methodology to the microarray expression dataset from 2009, and found that the marker 27B, which is the closest to Chr 2L: 6,250,000, included several genes that showed similar changes in expression in response to lead. To be specific, when we extracted all expression levels of the available microarray probes for the 89 genes identified by the current RNA-seq data and compared them to the hierarchical clustering heatmap at 27B, we found similar expression segregation patterns as previously. In the left panel (lead-treated) of Figure 6, genes could be divided into three groups: g1, g2, and g3 according to the similarity of the expression pattern. We noticed that most genes from g1 (10 out of 12, 83.3%) and g3 (29 out of 34, 85.3%) belong to RNA-seq G1 group (Figure 5), while most genes from g2 (20 out of 29, 70.0%) were the same as G2. The right panel of Figure 6 was created by keeping the order of the sample and associated genes but replacing lead-treated expression data with control ones.

Like the results of the RNA-seq data, the 2009 microarray results showed that the coherent expression patterns formed in lead-treated data were disrupted, and apparently made more random, in control data. By “coherent” we mean that the gene expression patterns in the lead data, but not the control data, cluster into blocks of higher-expressed genes or lower-expressed genes. In other words, the genes forming coherent expression patterns could only be observed at equivalent expression levels after lead exposure. This suggested that the segregated expression patterns at the trans-eQTL locus at 27B were found in both RNA-seq data and in microarray data.

However, the color-coded bar above the heatmap in the 2009 microarray data, indicating the original parent of origin at this 27B location (Figure 6), showed no significant difference based on parental origin. This indicates that two parental lines in the 2009 data—Oregon R and Russian 2B (which are not included in the eight parental lines used in the RNA-seq analyses), have no differential influence over associated gene expression profiles at the 27B (Chr 2L: 6,250,000) locus. This probably explains why this location was not detected as a *trans-*eQTL hotspot in the microarray experiment in the original experiments from 2009. This also indicates that eight-way analysis, which includes more genetic variation than two-way analysis, is more robust and likely includes more *trans*-eQTLs.

## Discussion

Here we investigated gene expression in *Drosophila* heads from 79 eight-way RILs to identify lead-responsive *cis-* and *trans-*eQTLs. We also went one step further to provide the additional evidence for the existence of the lead-responsive *trans-*eQTL hotspots. With the help of the clustering analyses, we confirmed that the expression traits of the progeny could be sub-grouped based on the genetic contributions of the parents.

There are several advantages of this eight-way eQTL analysis using RNA-seq compared with our previous two-way study by using Microarrays. First, although RNA-seq might have bias during alignment process (Munger et al., 2014), it avoids the possibility of false positive reads due to the limitation of the microarray technology. For example, a SNP at a probe site in one line but not another could be misinterpreted as differential expression in microarray data, but not in RNA-seq data (Xiao et al., 2002; Fadiel and Naftolin, 2003). Second, the advantage of RNA-seq lies in its independence to prior sequence knowledge. This enables the detection of structural variations such as alternative splicing and novel transcripts. And we successfully used this set of RNA-seq data to search for splicing QTLs (sQTLs)—QTLs that were associated with splicing events, both *cis*-sQTLs and *trans*-sQTLs (Ruden et al., 2017). Third, the abundant genotype information in RNA-seq data, which includes 11,768 underlying parental haplotype structures, makes it more likely to pinpoint the precise eQTL locus, while the previous microarray eQTL analysis only contain 92 genomic markers, each of which was at least 5 cM wide (Ruden et al., 2009). Fourth, this time we have more parental lines involved (eight-way vs. two-way), which should include more genetic variation that are present in *Drosophila* species.

Another criterion worth mentioning is the sample size. Because of the cost, we could only afford to analyze 79x2 RNA-seq samples (control and lead) with no replicates. We probably would have identified more *trans-*eQTL hotspots if we included more recombinant inbred lines or replicates. In general, with the cost being a major limiting factor, most investigators believe that more eQTL can be identified by increasing the number of recombinant inbred lines analyzed rather than increasing the number of replicates of each line. If one considers the unit of replication being a haplotype rather than a recombinant inbred line, then the number of replicates for each haplotype will be the number of times that haplotype was sequenced in the experiment. For example, if 100 recombinant inbred lines were sequenced, and two haplotypes are present for one locus, then each haplotype would be replicated approximately 50 times if the haplotype was randomly distributed in the lines.

There is some concern that our data does not overlap in some respects with a recently published study with some of the same DSPR lines. For example, the DSPR group mapped genome-wide expression variation in 2014 in their eight-way cross lines. They generated an eQTL interactive map and found two *trans-*eQTL hotspots. However, they did not have an exposure model, and the *trans-*eQTLs did not overlap with the hotspots identified in our study. This could be explained because their experiments included more genetic differences: heterozygotes from parental population groups A and B (both A1, A2 and B1, B2) (King et al., 2014), while we only considered a subset of homozygotes in one parental subgroup, A2. Furthermore, they worked with heterozygotes due to inbreeding depression (King et al., 2014). In contrast, our experiments used only A1–A8 and each of our RILs was a homozygous mosaic of the eight parental lines. The consistent finding is that most of the eQTLs were multiallelic (King et al., 2014), i.e., many of the genes with eQTLs are expressed at more than two different levels in the 16 founder lines, and the same phenomenon has been observed in our study.

In contrast to the DSPR group, we included developmental lead poisoning as a perturbation and searched for lead-responsive eQTLs. We have successfully identified lead-responsive *trans-*eQTL hotspots in our 2009 study. We found that some *trans-*eQTL hotspots were formed in response to lead poisoning and some *trans-*eQTL hotspots disappeared after lead treatment. The clustering analysis has shown the samples from different parental genetic origins responded differently in downstream gene expression profiles before or after lead exposure.

One of our most important findings is that the differential gene expression pattern upon Pb exposure found in the RNA-seq data was replicated in the microarray data. And for the two groups of genes, G1 was mostly Pb-specific eQTLs and their functions were linked with neuronal and response to stimuli, while G2 was mostly metabolic-related stable eQTLs. Previous papers have hypothesized that gene expression profiling patterns associated with *trans-*eQTL hotspots reflect biological pathways (Wu et al., 2008); however, in this hotspot, we did not ended up with any enriched pathways among the associated genes.

At the *trans*-eQTL hotspot we focused on in this paper at 27B, we found a conserved nucleotide motif among the regulated genes' promoter sequences—AAAAAYA (C: C or T). This motif matches corresponds to the binding site for the transcription factor Hunchback. Our next steps will include identifying and knocking down candidate genes responsible for the *trans-*eQTL hotspots, such as *hb*, and determining whether the expression levels of the proposed downstream genes are influenced. Such “perturbation analyses” are needed to validate candidate *trans*-eQTLs (Yvert et al., 2003).

Detailed analyses of the *cis-*eQTL, such as allele-specific expression (ASE) and transposase-accessible chromatin using sequencing (ATAC-seq) analyses of brains from heterozygous flies after Pb exposure (Buenrostro et al., 2015), are in progress and will be presented in a future paper. Also in progress in our laboratory are single-cell RNA-seq (scRNA-seq) experiments of Drosophila brains after lead exposure. Recently, scRNA-seq experiments with Drosophila brains have identified over 50 brain cell types (Croset et al., 2018; Davie et al., 2018). We anticipate that scRNA-seq of Drosophila brains, as well as other model organisms such as mouse (Zeisel et al., 2015; Karlsson and Linnarsson, 2017; Rosenberg et al., 2018), will identify specific neuronal and glial cell types that are most deleteriously affected by Pb exposure. We also plan to include behavioral data to determine if the differential expression changes in different parental strains could provide a protective mechanism to respond to lead poisoning. We acknowledge that both the current and the previous studies used a chronic lead exposure throughout larval development to the adult stage and that many, if not most, of the gene expression changes could reflect global “re-organization” of transcription in the adult head that reflects events very much downstream of the actual mode of action of the toxicant. To address this issue, future studies will also investigate the effects of acute effects of lead on gene expression and chromatin organization.

In conclusion, RNA-seq technology is a powerful tool in obtaining genome-wide expression profiles and identifying *cis-*and *trans-*eQTLs in *Drosophila*. The hierarchical clustering analyses display the expression patterns of the eQTL-associated genes and show that they segregate by genotype. We have successfully made progress in understanding how *trans-*eQTL hotspots alter the genomic/transcriptomic response to Pb exposure. This might help us understand downstream events that cause Pb-induced toxicity, thus opening a gate toward understanding the neurotoxicity of lead.

## Author contributions

DR supervised the research from the beginning to the end. WQ conducted the experiments and performed all of the bioinformatics analyses. KG supervised the DNA sequencing and alignments and some of the bioinformatics. RP-R supervised the bioinformatics.

### Conflict of interest statement

The authors declare that the research was conducted in the absence of any commercial or financial relationships that could be construed as a potential conflict of interest.
