# Supplementary material for: Lead Modulates trans- and cis-Expression Quantitative Trait Loci (eQTLs) in Drosophila melanogaster Heads
Source: Front Genet. 2018 Sep 20;9:395. doi: 10.3389/fgene.2018.00395 (PMC6158337; doi:10.3389/fgene.2018.00395)
Supplement: Supplementary file 1 [file Data_Sheet_1.pdf]

SUPPLEMENTARY INFORMATION

Lead Modulates *trans-* and *cis*-expression Quantitative Trait Loci (eQTLs) in *Drosophila melanogaster* Heads

Wen Qu [*], Katherine Gurdziel [†], Roger Pique-Regi [†, ‡], Douglas M. Ruden[*,†, §]

Affiliations: *Department of Pharmacology, Wayne State University, Detroit, MI 48201,

†Department of Obstetrics and Gynecology, Wayne State University, Detroit, MI 48201, ‡Center for Molecular Medicine and Genetics, Wayne State University, Detroit, MI 48201, and §Institute of Environmental Health Sciences, Wayne State University, Detroit, MI 48201

Corresponding Author: Dr. Douglas Ruden

Address: Director of Epigenomics, Department of Obstetrics and Gynecology, Institute of Environmental Health Sciences (IEHS), C.S. Mott Center for Human Health and Development, 275 E. Hancock Ave., Room 002, Detroit, MI 48201.

Phone: 313-577-6688

Fax: 313-577-0082

Email: douglas.ruden@gmail.com

Running Title: Lead eQTLs in *Drosophila* Heads

Supporting Information

S1 Fig. Lead Treatment Altered the Gene Expression Levels in *Drosophila* Model. MA plots for change in gene expression (n=2698) comparing lead-treated (n=79) with control-treated samples (n=79). M= log2(P/C), A= (log2(C)+log2(P))/2. Red dots: genes expression profiles were not significantly changed; Cyan dots: genes expressions were significantly changed (0.214 ± 0.223 mean log2 fold changes +- s.d, FDR<0.0001). P: lead-treated FPKM values; C: control FPKM values.

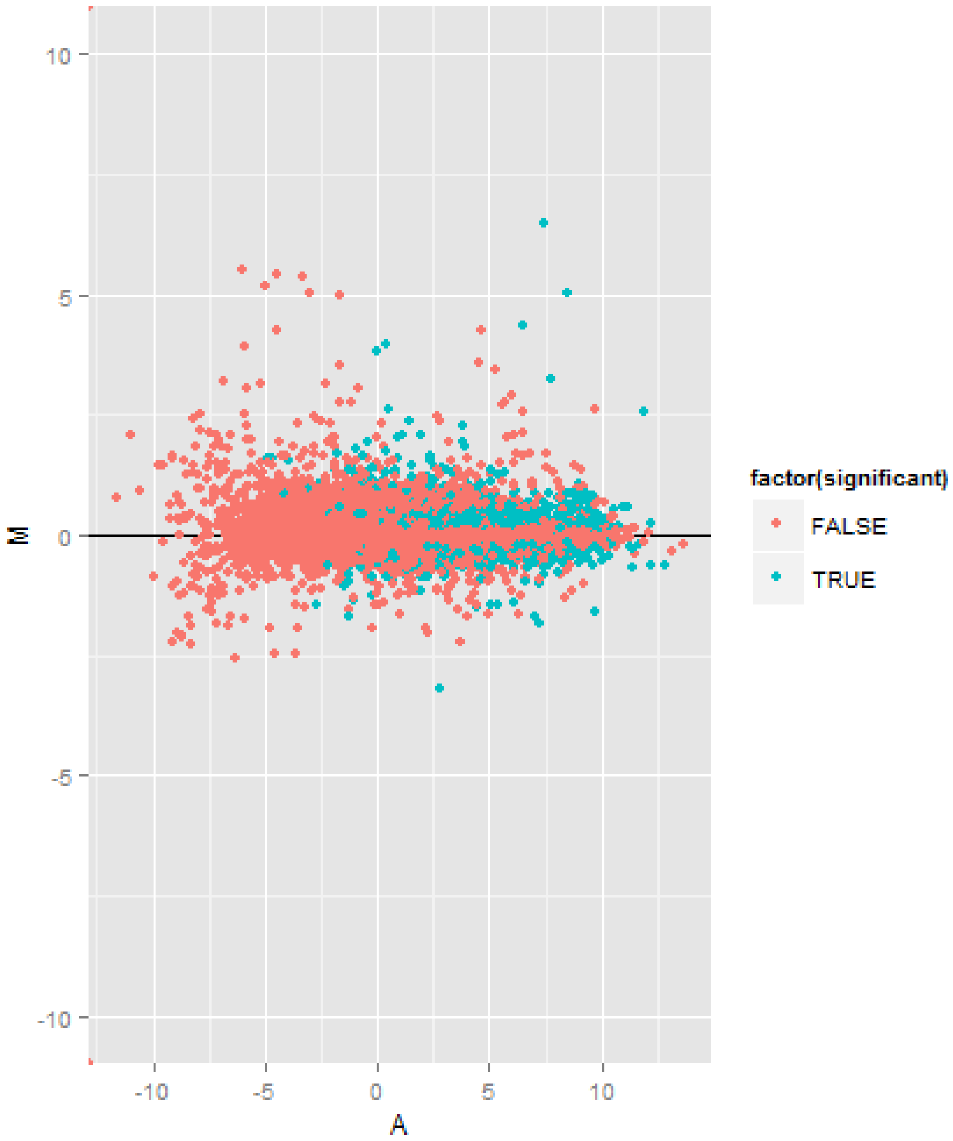

S2 Fig. Gene ontology enrichment analysis of lead treatment in *Drosophila melanogaster* male head samples. Gene Ontology (http://geneontology.org/) was used to detect over represented GO categories in RNA-seq data (FDR <0.0001). Y-axis shows the logarithm of each significant GO ID's *p*-value (after Bonferroni correction for multiple testing). Significant GO IDs among upregulated genes after lead exposure were colored in red and GO ID among downregulated genes in green. GO IDs related with synapses and neuronal functions were highlighted in **bold**.

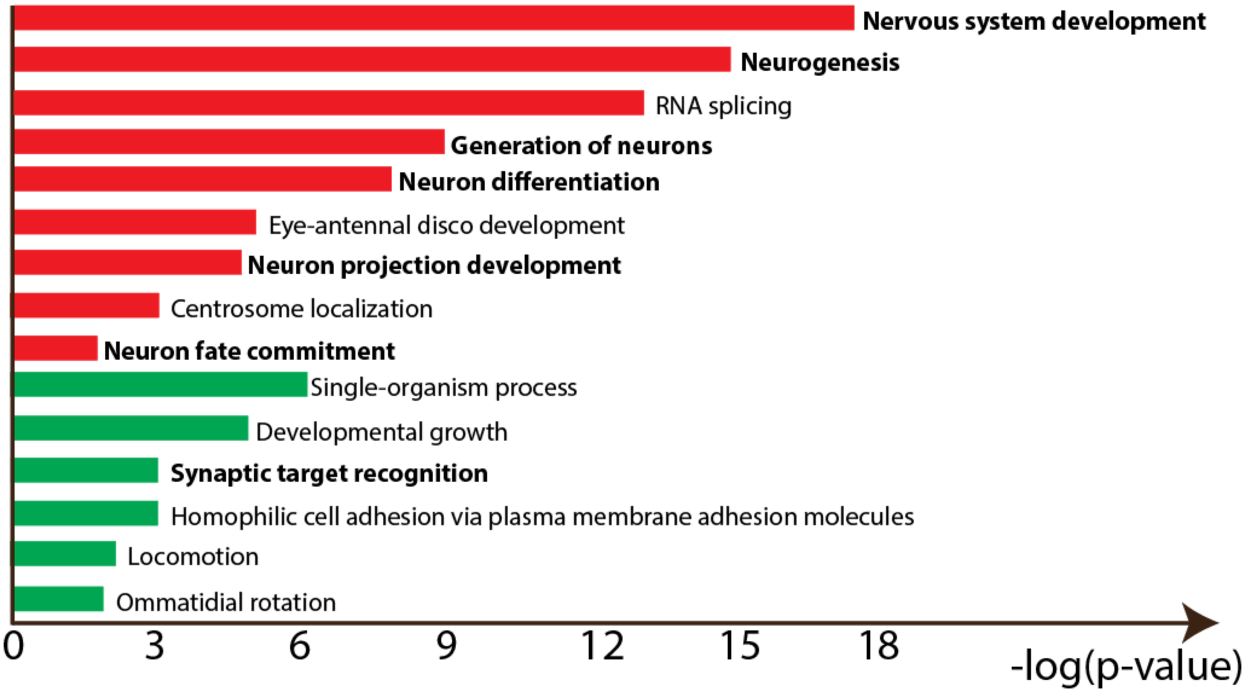

S3 Fig. Venn diagrams demonstrating the overlaps between control eQTLs and lead-treated eQTLs. (A) gene numbers for *cis*-eQTLs. (B) gene numbers for *trans*-eQTLs.

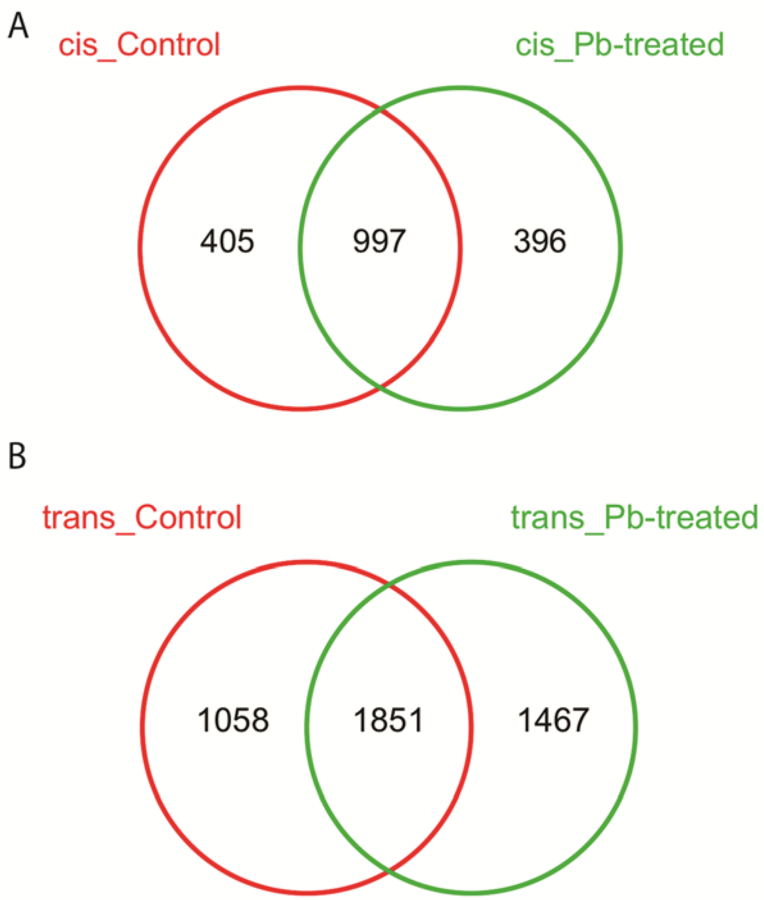

S4 Fig. Examples of *trans*-eQTL associated genes. (A) one example of lead-specific eQTL in G1 family. In the left two panels, the x-axis represents the *Drosophila* genomic locations and y-axis represents the LOD score of the gene. The red horizontal line indicates the threshold for *p*-value to be 0.05 after 1000 permutation test. The green dash vertical line indicates the location of the gene. (B) another example of lead-specific eQTLs in G1 family. (C) one example of stable eQTLs. (D) another example of stable eQTLs. (E) an example of the Pb-specific eQTLs in G1 family.

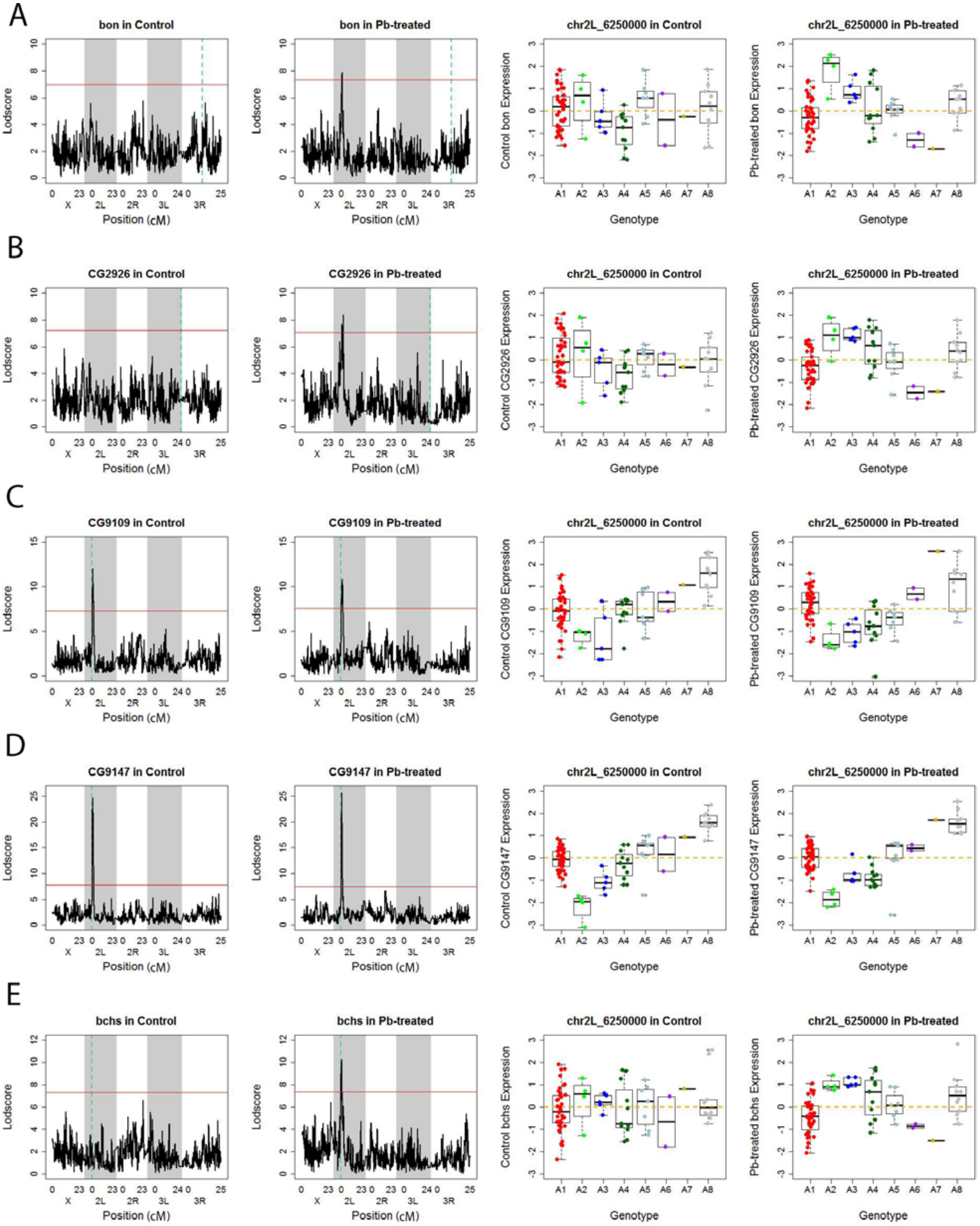

A
bon in Control
bon in Pb-treated
chr2L_6250000 in Control
chr2L_6250000 in Pb-treated
Control bon Expression
Pb-treated bon Expression
B
CG2926 in Control
CG2926 in Pb-treated
chr2L_6250000 in Control
chr2L_6250000 in Pb-treated
Control CG2926 Expression
Pb-treated CG2926 Expression
C
CG9109 in Control
CG9109 in Pb-treated
chr2L_6250000 in Control
chr2L_6250000 in Pb-treated
Control CG9109 Expression
Pb-treated CG9109 Expression
D
CG9147 in Control
CG9147 in Pb-treated
chr2L_6250000 in Control
chr2L_6250000 in Pb-treated
Control CG9147 Expression
Pb-treated CG9147 Expression
E
bchs in Control
bchs in Pb-treated
chr2L_6250000 in Control
chr2L_6250000 in Pb-treated
Control bchs Expression
Pb-treated bchs Expression
Lodscore
Position (cM)
X 2L 2R 3L 3R
Genotype
A1 A2 A3 A4 A5 A6 A7 A8

S5 Fig. Common motif shared by the associated genes at the Chr 2L: 6,250,000 *trans*-eQTL hotspot. (A) The distribution and frequencies of each basepairs. (B) The nucleotide distribution matrix shows the nucleotide frequencies observed in aligned binding sites of the corresponding transcription factor. Basepairs in red indicate a high information content, which means the matrix exhibits a high conservation (> 60%) at this position. Genomatix made the basepairs in capital letters denote the core sequence used by MatInspector. The core sequence of a matrix is defined as the (usually 4) highest conserved, consecutive positions of the matrix. (C) Common motif of the associated genes at the *trans*-eQTL hotspot. (D) The common motif detected in (C) resembles hb with *p*-value to be 8.11e-04. (E) - (F): The LOD score plot of hunchback.

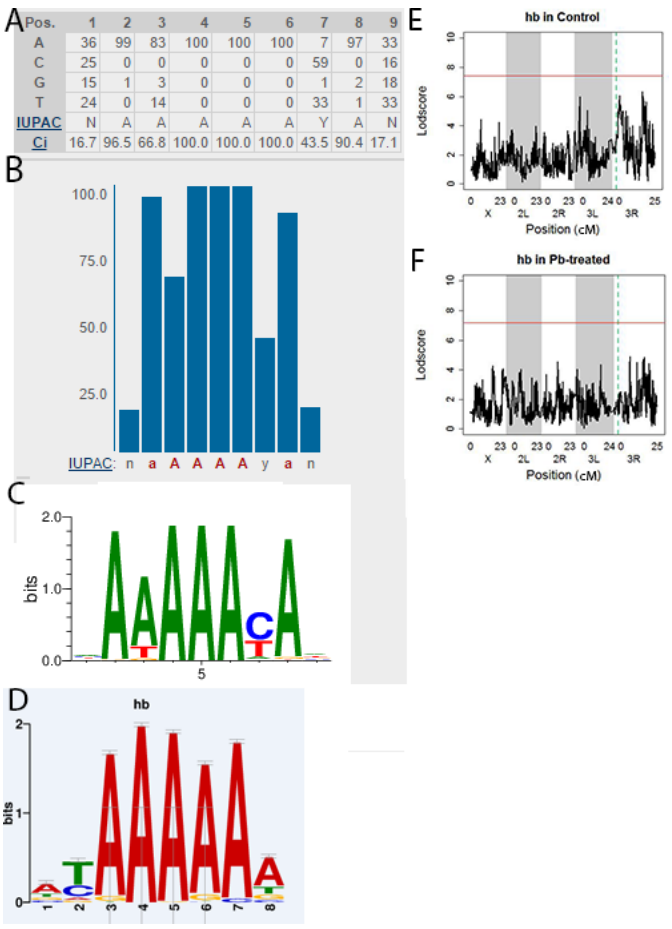

Pos.	1	2	3	4	5	6	7	8	9
A	36	99	83	100	100	100	7	97	33
C	25	0	0	0	0	0	59	0	16
G	15	1	3	0	0	0	1	2	18
T	24	0	14	0	0	0	33	1	33
IUPAC	N	A	A	A	A	A	Y	A	N
Ci	16.7	96.5	66.8	100.0	100.0	100.0	43.5	90.4	17.1

S6 Fig. *Trans*-eQTL hotspots targeted after SVA. Numbers of significant associated genes identified after SVA process (*p*-value ≤ 0.05). The lead-responsive *trans*-eQTL hotspot at Chr 2L: 6,250,000 (red dashed line) was still one of the strong peaks after SVA processing.

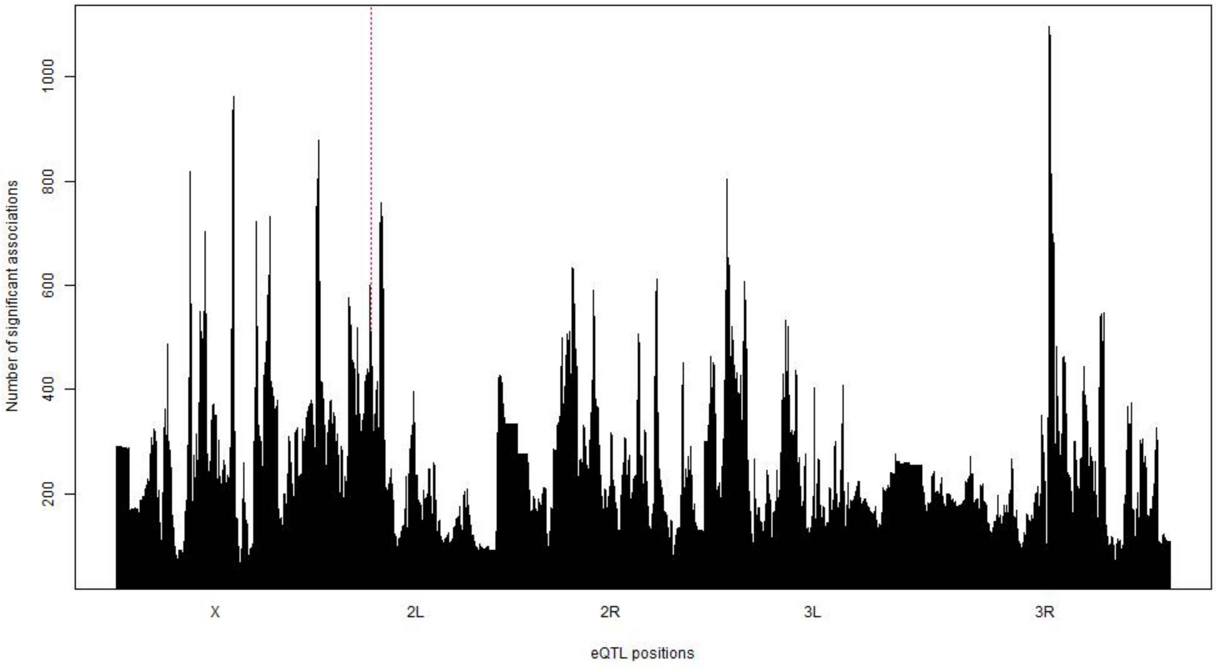

S1 Table. Detailed information about the lead-responsive *trans*-eQTL hotspots (*p*-value ≤ 0.00001 with the distribution generated by 1000 permutations).

#No	status	Chr	Range (Mb)	Length (Mb)	peak location (Mb)	#genes @ peak
1	Ctrl	2L	2.61	0.01	2.61	42
2	Ctrl	2R	1.51-1.56, 1.58, 1.60-1.67, 1.71-1.73	0.18	1.60	46
3	Ctrl	2R	4.25-4.53, 4.59-4.61, 4.70-4.72, 4.78	0.33	4.41, 4.50	49
4	Ctrl	2R	13.45-13.53	0.09	13.49	44
5	Ctrl	3R	4.90-5.14, 5.29-5.73	0.70	5.58	62
6	Ctrl	3R	11.34-11.61, 11.82-11.96, 12.62-12.76	0.58	11.44, 11.49-11.54, 11.83	45
7	Pb	2L	5.96, 6.11-6.41, 7.00-7.07	0.40	6.25	89
8	Pb	2L	10.89-10.90, 10.93	0.03	10.89, 10.90, 10.93	39
9	Pb	2L	16.79-16.80, 16.82	0.03	16.79-16.80, 16.82	39
10	Pb	2R	3.83- 3.94, 4.06, 4.08, 4.20- 4.79, 4.82-4.84, 4.89, 4.99-5.12, 5.22-5.27, 5.37	0.99	4.51, 4.57	51
11	Pb	2R	8.64, 9.26-9.32, 9.34-9.37, 9.40-9.42, 9.45	0.16	9.32	63
12	Pb	3R	5.42-5.75, 5.77-5.78, 5.82	0.37	5.59	61

13	Pb	3R	9.74-9.75, 9.88-10.05, 10.24-10.25, 10.30-10.49, 10.52-10.73, 11.10-11.14, 11.18-11.27, 11.32-11.37, 11.39-11.68, 11.70-12.16, 12.20-12.21, 12.60-12.64, 12.73, 12.75-12.76	1.70	12.03	53
14	Pb	3R	21.47-21.48	0.02	21.48	42

S2 Table. (A) GO function categories for the associated genes at Chr 2L: 6,250,000. (B) eQTL types for genes at G1 and G2.

(A)

Group	Neuron-related	Metal ion binding	Response to stimuli, immune system, cell death, DNA damage	Other metabolic processes	Behavior (mating, etc.)	Unknown function	total
G1	18 (30%)	7 (11%)	12 (20%)	13 (21%)	1 (1%)	10 (16%)	61
G2	1 (3%)	1 (3%)	2 (7%)	18 (64%)	1 (3%)	5 (18%)	28
total	19 (21%)	8 (9%)	14 (16%)	31 (35%)	2 (2%)	15 (17%)	89

(B)

Group	eQTL at Chr 2L: 6,250,000 both in control & lead-treated	eQTL at Chr 2L: 6,250,000 only in lead-treated	eQTL at Chr 2L: 6,250,000 only in lead-treated	others	total
G1	3 (5%)	52 (85%)	2 (3%)	4 (6%)	61
G2	22 (78%)	4 (14%)	2 (7%)	0	28
total	25 (28%)	56 (63%)	4 (4%)	4 (4%)	89

S3 Table. Experimental design and Result Comparison between the microarray in 2009 and RNA-seq in 2012.

	Microarray in 2009			RNA-seq in 2012		
Genotypes	two-way			eight-way		
Genomic information	SNPs			Genomic origins		
Numbers of Genomic locations	92 (markers)			11768		
Numbers of Samples in each condition	75			79		
Numbers of Genes detected	~14000 (18,952 probesets)			13381		
Condition	mixing 250μM lead acetate in the fly food as lead exposure					
Sample collected	Whole male *Drosophila*			Male *Drosophila* head		
Technique used	Microarray			RNA sequencing		
The criteria for significant eQTLs	The 1000 permutation LOD scores have a *p*-value of less than 0.0001			The 1000 permutation LOD scores have a *p*-value of less than 0.05		
Definition of *cis*-eQTL	Significant eQTLs within a 5 cM sliding window			Significant eQTLs within 1 cM		
Definition of *trans*-eQTL	Significant eQTLs outside the 5 cM sliding window			Significant eQTLs outside the 1 cM		
Definition of *trans*-eQTL hotspots	96 probesets in a 5cM window			38 (Ctrl), 41 (Pb) genes in a 1 cM window (*p*-value <0.00001)		
	Control-only	Overlap	Lead-Only	Control-only	Overlap	Lead-Only
Numbers of *cis*-eQTLs detected*	405	440	544	405	997	396
Numbers of *trans*-eQTLs detected*	948	357	1191	1058	1851	1467

*Please note that the numbers of *cis*- and *trans*- eQTL detected in microarray assay or in RNA-seq assay are not comparable due to differential genomic information.
